# Cardiac disease and prognosis associated with ventricular tachyarrhythmias in young adults and adults

**DOI:** 10.1186/s12872-022-02552-6

**Published:** 2022-03-31

**Authors:** Kathrin Weidner, Michael Behnes, Tobias Schupp, Jorge Hoppner, Ibrahim El-Battrawy, Uzair Ansari, Ahmad Saleh, Gabriel Taton, Linda Reiser, Armin Bollow, Thomas Reichelt, Dominik Ellguth, Niko Engelke, Thomas Bertsch, Dirk Große Meininghaus, Ursula Hoffmann, Ibrahim Akin

**Affiliations:** 1 Department of Cardiology, Angiology, Haemostaseology and Medical Intensive Care, University Medical Centre Mannheim, Medical Faculty Mannheim, Heidelberg University, Heidelberg, Germany; 2 European Center for Angio Science (ECAS) and German Center for Cardiovascular Research (DZHK) Partner Site Heidelberg/Mannheim, Mannheim, Germany; 3 Department of Nuclear Medicine, University Hospital Heidelberg, Heidelberg, Germany; 4 Institute of Clinical Chemistry, Laboratory Medicine and Transfusion Medicine, Nuremberg General Hospital, Paracelsus Medical University, Nuremberg, Germany; 5 Department of Cardiology, Carl-Thiem-Klinikum Cottbus, Cottbus, Germany

**Keywords:** Young adults, Age, Ventricular tachyarrhythmia, Cardiovascular risk factors, Coronary artery disease, Long-term mortality, Cardiac-related disease

## Abstract

**Background:**

This study evaluates cardiac diseases and prognosis in young adults and adults presenting with ventricular tachyarrhythmias (VTA).

**Methods:**

The present longitudinal, observational, registry-based, monocentric cohort study includes all consecutive patients 45 years old or younger presenting with VTA at admission from 2002 to 2016. Rates of coronary angiography, coronary artery disease (CAD) and need for percutaneous coronary intervention (PCI), cardiac diseases associated with VTA, and differences in long-term prognostic endpoints for young adults (20–34 years old) were analyzed and compared to those of adults (35–45 years old), for whom multivariable risk prediction models were developed. Kaplan–Meier analyses were performed according to age and type of VTA.

**Results:**

A total of 259 consecutive patients were included in the study (36% young adults and 64% adults). At admission, 38% of young adults had VTA due to CAD that required PCI. Furthermore, VTA in young adults was commonly idiopathic (27%), or had underlying channelopathies (18%), primary cardiomyopathies (13%) or acute myocardial infarction (AMI, 11%). In adults, VTA was mostly associated with AMI (28%), though the rate of idiopathy was still high (20%). A total 41% of all patients received cardiopulmonary resuscitation (CPR), for whom AMI (STEMI 17%, NSTEMI 24%) was most frequently observed. Irrespective of the type of VTA, all-cause mortality was similar for young adults and adults. In young adults, left ventricular ejection fraction (LVEF) < 35% (HR = 33.590) was associated with increased long-term all-cause mortality.

**Conclusion:**

Despite high rates of idiopathic ventricular tachyarrhythmias, CAD and AMI are common causes of VTA and CPR in adults 45 years old and younger. Young adults and adults had comparable survival at index hospitalization and after 2.5 years irrespective of the type of VTA.

*Clinical trial registration* clinicaltrials.gov identifier: NCT02982473.

## Introduction

Ventricular tachyarrhythmias (VTA) and sudden cardiac death (SCD) are fatal diseases occurring across all age groups. However, there is insufficient data to investigate their impact on young adults in the Western world [1, 2]. SCD in patients 45 years old or younger is rare, with a prevalence of only 5% [1, 2]. Regarding the years of potential life lost, SCD in the young is a devastating phenomenon and a significant health problem [2]. It is widely believed that most young SCD victims suffer from hypertrophic cardiomyopathy (HCM) [3, 4]. However, most studies investigated young competitive athletes, who do not reflect the general young population [3, 4]. Therefore, SCD and underlying coronary artery disease (CAD) among young adults are presumed to be more common than expected [2, 4]. Recent studies have conflicted about the potential impact of the cardiovascular risk factors (CVRF) arterial hypertension, diabetes mellitus, smoking, hyperlipidemia and obesity in young SCD victims. Additionally, most studies have included patients presenting with out-of-hospital cardiac arrest (OHCA) irrespective of the underlying cause of SCD, thereby evaluating various comorbidities, such as trauma, respiratory failure, or drug overdose, and complicating further comparative in-depth analyses and data interpretation [4, 5]. Limited data are available for investigations of young adult patients with presumed cardiovascular causes of SCD [2]. More detailed studies on specifically preventable causes, including CVRF and CAD, are essential to guide future studies on improving risk prediction and effective prevention of SCD in young adults [1].

Therefore, the present longitudinal, observational, registry-based, monocentric cohort study retrospectively investigates the rates of CAD, underlying cardiac diseases, differences in prognostic endpoints, and prediction models in consecutive patients presenting with ventricular tachyarrhythmias.

## Methods

### Study patients, design, and data collection

The present study retrospectively included all consecutive patients with ventricular tachyarrhythmias (VTA) from 2002 until 2016 at one institution, as previously published [6, 7]. Some of these VTA patients suffered OHCA. Patients who survived out-of-hospital cardiopulmonary resuscitation (CPR) were included in the study. Accordingly, patients who passed away following OHCA or those who were not transferred to the heart center were excluded. In-hospital cardiac arrest (IHCA) of VTA patients during the index hospitalization was also documented. All clinical data related to the index event was documented using patient files, daily records, diagnostic examinations and laboratory values, electrocardiograms (ECG), and device recordings. All additional information was derived from the electronic hospital information system.

VTA comprise ventricular tachycardia (VT) and ventricular fibrillation (VF), as defined by current international guidelines [8]. Sustained VT (SVT) is defined as lasting more than 30 s, or by additional hemodynamic collapse within 30 s. Non-sustained VT (NSVT) is defined as lasting less than 30 s. VT indicates a wide QRS complex (≥ 120 ms) at a rate greater than 100 beats per minute. VTA were documented in the present study by 12-lead ECG, ECG telemonitoring, and implantable cardioverter-defibrillator (ICD). In the case of an unstable course or during CPR, documentation was performed with external defibrillator monitoring. Documented VF was treated with external defibrillation and, in the case of prolonged instability, with additional intravenous anti-arrhythmic drugs during CPR [6, 7].

The documented data also included baseline characteristics, prior medical history, prior medical treatment, length of index hospitalization, detailed findings of laboratory values at baseline, data derived from all noninvasive or invasive cardiac diagnostics, and device therapies. Device therapies included coronary angiography, electrophysiological examination, previously or newly implanted ICDs, pacemakers, or cardiac contractility modulation already implanted at index hospitalization or at follow-up. Imaging modalities included echocardiography or cardiac magnetic resonance imaging (cMRI). Overall presence of an activated ICD refers to patients with an ICD implanted before admission, patients who received a new ICD at index hospitalization, and patients who received an ICD in the period between index hospitalization and follow-up. ICD types include sole ICD, subcutaneous ICD (s-ICD), and cardiac resynchronization therapy defibrillator (CRT-D). Pharmacological treatment was documented according to the discharge medication of patients surviving index hospitalization. Rates of overall ICDs and pharmacological therapies were determined by the number of surviving patients discharged from the index hospitalization [6, 7].

The documentation period for each patient lasted from their index hospitalization until 2016. Independent cardiologists blinded to final data analyses performed documentation of all medical data during the patients’ individual periods of hospitalization.

The present study is derived from an analysis of the “Registry of Malignant Arrhythmias and Sudden Cardiac Death-Influence of Diagnostics and Interventions (RACE-IT).” It describes a retrospective, single-center registry of consecutive patients with ventricular tachyarrhythmias and SCD admitted between 2002 and 2016 to acute care in the University Medical Center Mannheim (UMM), Germany (clinicaltrials.gov identifier: NCT02982473). The registry was established according to the principles of the Declaration of Helsinki. It was approved by the Ethics Committee II of the Faculty of Medicine Mannheim, University of Heidelberg, Germany.

### Definition of study groups and inclusion and exclusion criteria

For the present analysis, risk stratification was performed according to age. Young adults (20–34 years old) were compared to adults (35–45 years old). Patients younger than 20 years old or older than 45 years old were excluded. Each patient was counted only once for inclusion when presenting with the first episode of VTA. In accordance with prior studies, the age of 45 was chosen as the upper cutoff to maximize the capture rates of heritable cardiac syndromes and prevent overlap with CAD [1].

### Definition of underlying cardiac diseases

Patients with acute myocardial infarction (AMI) included those with ST-segment elevation myocardial infarction (STEMI) and non–ST-segment elevation myocardial infarction (NSTEMI). According to current guidelines, AMI was defined as the presence of an acute myocardial injury with clinical evidence of acute myocardial ischemia and with detection of a rise or fall of cTn values, with at least one value above the 99^th^ percentile upper reference limit. At least one of the following conditions must exist: symptoms of myocardial ischemia, new ischemic ECG changes, development of pathological Q waves, imaging evidence of new loss of viable myocardium or new regional wall motion abnormality in a pattern consistent with an ischemic etiology, or identification of a coronary thrombus by angiography [7, 9, 10]. The results of coronary angiography at index hospitalization were retrieved to update the CAD diagnosis in these patients [8, 9].

Primary cardiomyopathies included nonischemic cardiomyopathies which were defined as left ventricular ejection fraction (LVEF) < 55% in the absence of CAD and valvular or congenital heart disease sufficient to cause the observed myocardial abnormality. The following were defined as primary cardiomyopathies: dilated cardiomyopathy (DCM), nonobstructive HCM, arrhythmogenic right ventricular dysplasia (ARVD), noncompaction cardiomyopathy, and restrictive cardiomyopathy [8, 11, 12].

Secondary cardiomyopathies were defined as systemic multiorgan disorders with pathological myocardial involvement [12]. Storage disorders (e.g., Morbus Fabry, amyloidosis, cardiac glycogenosis, cardiac hemochromatosis, Morbus Gaucher), systemic inflammatory disease (sarcoidosis), septic cardiomyopathy, and postpartum cardiomyopathy were included as secondary cardiomyopathies [12].

Patients with ischemic cardiomyopathy (ICMP) included those with LVEF < 55% and relevant CAD (either in documented history or newly diagnosed), as well as patients with present or history of AMI. The results of coronary angiography at index hospitalization were used to update the CAD diagnosis in these patients [8, 12].

Channelopathies were defined as disorders resulting from a dysfunction of ion channels located in the membranes of a cell and cellular organelles. Defects in the ion channels were caused either by genetic or acquired factors. Channelopathies included Brugada syndrome, long QT syndrome, and short QT syndrome, according to current guidelines [8, 12, 13].

Toxic cardiomyopathy was defined as cardiac dysfunction caused by acute drug intoxication after abusing cocaine, amphetamines, heroin, cannabis, tricyclic antidepressants, benzodiazepines, or alcohol. However, the current literature still lacks a precise definition and classification of toxic cardiomyopathy [12].

Congenital disorders were defined as heart diseases caused by defects in cardiac anatomy or vessels which were present at birth [14].

Other causes of VTA were pulmonary embolism, high-voltage accident, decompensated liver cirrhosis with hypokalemia, respiratory failure related to obstructive pulmonary diseases, chronic diarrhea with consecutive hypokalemia, Wolff-Parkinson-White (WPW) syndrome; fast, broad, and irregular (FBI) tachycardia; and perioperative causes during orthopedic and gynecological surgery and surgical oncology.

Patients were classified as idiopathic when results for laboratory tests, echocardiography, electrophysiological examination, coronary angiography, and cMRI were inconclusive.

Unexplained was defined as patients with ventricular tachyarrhythmias who died immediately after hospitalization in the emergency room, or during the first 24 h in the ICU without further diagnostic.

### Study endpoints

The primary endpoint was all-cause mortality at a long-term follow-up of 2.5 years. Secondary endpoints were cardiac death at 24 h, all-cause mortality at index hospitalization, all-cause mortality after discharge, and the composite arrhythmic endpoint at 2.5 years, which consisted of cardiac death at 24 h, recurrent VTA, and appropriate ICD therapies. Cardiac death occurring within 24 h of hospital admission was defined either as cardiac death after the onset of VTA or an assumed unstable cardiac condition on admission [8].

All-cause mortality was documented with our electronic hospital information system and by directly contacting state resident registration offices (“bureau of mortality statistics”) across Germany. All-cause mortality comprised all kinds of death occurring within the periods of time defined in the paragraph above. Patient identity was verified by name, surname, date of birth, and registered living address. Lost to follow-up rate was 1.7% (n = 48) regarding survival until the follow-up period.

### Statistical methods

Quantitative data are presented as mean ± standard error of mean, median and interquartile range, and ranges, depending on the data distribution; they were compared using the Student’s *t*-test for normally distributed data and the Mann–Whitney *U* test for nonparametric data. The Kolmogorov–Smirnov test tested deviations from a Gaussian distribution. Spearman’s rank correlation for nonparametric data was used to test univariate correlations. Qualitative data are presented as absolute and relative frequencies and compared using the chi-square test or the Fisher’s exact test, as appropriate. Multivariate Cox regression models were developed using the “forward selection” option, where only statistically significant variables (p < 0.05) were included and analyzed simultaneously (see below). Rates of coronary angiography, CAD, and PCI were analyzed according to predefined age categories: 20–24, 25–29, 30–34, 35–39, and 40–45 years.

A binary age classification was made based on recent literature defining young adults as 20–34 years old and adults as 35–45 years old [1, 15]. Underlying cardiac diseases for VTA and CPR were present in both subgroups. Univariate prognostic differences of the primary and secondary prognostic endpoints were analyzed using the Kaplan–Meier method with log-rank comparisons and hazard ratios (HR) with 95% confidence intervals.

Multivariate Cox regressions were applied in both subgroups. Predefined variables used for multivariate Cox regressions included male gender, chronic kidney disease (CKD) (glomerular filtration rate < 60 mL/min/1.73 m^2^), LVEF < 35%, AMI (STEMI, NSTEMI), atrial fibrillation, and underlying ventricular tachyarrhythmias (VT or VF). The result of a statistical test was considered significant for p < 0.05. SAS, release 9.4 (SAS Institute Inc., Cary, NC, USA) was used for statistics.

## Results

### Study population

A total of 259 consecutive patients aged 20–45 years old and presenting with VTA at index hospitalization were included for the present study. Of these, 36% were young adults aged 20–34 years old, and 64% were adults aged 35–45 years old. As outlined in Table 1, adults showed higher rates of CVRF than young adults, such as arterial hypertension (26% vs. 8%, *p* = 0.001), hyperlipidemia (19% vs. 7%, *p* = 0.008), and smoking (34% vs. 22%, *p* = 0.037). In prior medical history, adults had a higher rate of CKD (33% vs. 17%, *p* = 0.007). Accordingly, they were treated more frequently at discharge with angiotensin-converting enzyme inhibitors and angiotensin receptor blockers (46% vs. 24%, *p* = 0.001) (Table 1). Adults were also treated more frequently with statins (38% vs. 11%, *p* = 0.001) (Table 1).

**Table 1:** Clinical characteristics

Characteristic	Young adults 20–34 years old (n = 92; 36%)	Adults 35–45 years old (n = 167; 64%)	*p* value
Males, n (%)	53 (58)	118 (71)	**0.034**
Ventricular tachyarrhythmias, n (%)			
Ventricular tachycardia	54 (59)	79 (47)	
Non-sustained VT	27 (50)	47 (60)	0.279
Sustained VT	27 (50)	32 (40)	
Ventricular fibrillation	38 (41)	88 (53)	0.079
Cardiopulmonary resuscitation, n (%)	29 (31)	76 (45)	**0.028**
In-hospital	15 (16)	57 (34)	**0.009**
Out-of-hospital	14 (15)	19 (11)	
Cardiovascular risk factors, n (%)			
Arterial hypertension	7 (8)	44 (26)	**0.001**
Diabetes mellitus	3 (3)	13 (8)	0.148
Hyperlipidemia	6 (7)	31 (19)	**0.008**
Smoking	20 (22)	57 (34)	**0.037**
Cardiac family history	14 (15)	34 (20)	0.308
Prior medical history, n (%)			
Prior heart failure	4 (4)	16 (10)	0.131
Atrial fibrillation	6 (7)	19 (11)	0.205
Prior stroke	0 (0)	2 (1)	0.540
COPD	0 (0)	1 (0.6)	1.000
Chronic kidney disease	16 (17)	54 (33)	**0.007**
Laboratory data, mean ± SEM			
Hemoglobin [g/dl]	13.7 ± 2.2	14.1 ± 1.7	0.093
Potassium [mg/dl]	4.1 ± 0.1	4.3 ± 0.1	0.603
Creatinine [mg/dl]	1.0 ± 0.0	1.7 ± 0.4	0.093
Medication at discharge, n (%)			
Beta blocker	48 (58)	89 (65)	0.290
ACE inhibitor/ARB	20 (24)	63 (46)	**0.001**
Aldosterone antagonist	4 (5)	10 (7)	0.465
Digitalis	1 (1)	7 (5)	0.134
Amiodarone	4 (4)	6 (4)	0.879
Statin	9 (11)	52 (38)	**0.001**
Patients discharged alive, n (%)	83 (90)	137 (82)	0.078
Hospitalization time in days, median (IQR)			
Total hospitalization time	6 (1–52)	8 (1–55)	0.410
ICU time	1 (0–28)	2 (0–31)	0.523
Follow-up time in days, mean; median (range)	2217; 2338 (0–4608)	2039; 1931 (9–5095)	0.262

Bold type indicates statistical significance *p* < 0.05

ACE, angiotensin-converting enzyme; ARB, angiotensin receptor blocker; COPD, chronic obstructive pulmonary disease; ICU, intensive care unit; IQR, interquartile range; VT, Ventricular tachycardia

As shown in Table 1, adults had higher rates of overall CPR (45% vs. 31%, p = 0.028), prior CAD (12% vs. 4%, p = 0.043), and prior AMI (10% vs. 1%, p = 0.008). Furthermore, adults showed higher rates of AMI (STEMI: 12% vs. 4%, p = 0.018; NSTEMI: 16% vs. 7%, p = 0.034) and underwent coronary angiography at index hospitalization more often (58% vs. 28%, p = 0.001). CAD with coronary multi-vessel disease was more common in adults (p = 0.003) than in young adults. In young adults, 38% underwent coronary angiography that revealed CAD. All of these patients required PCI. In adults, 53% of patients undergoing coronary angiography showed CAD, and 84% needed PCI. Adults and young adults were equally likely to have an ICD at discharge (38% vs. 38%, p = 0.959) (Table 2).

**Table 2:** Cardiac comorbidities at index hospitalization

Characteristic	Young adults 20–34 years old (n = 92; 36%)	Adults 35–45 years old (n = 167; 64%)	*p* value
Left ventricular function, n (%)			
LVEF > 55%	33 (36)	57 (34)	0.188
LVEF 45–55%	14 (15)	17 (10)	
LVEF 35–44%	11 (12)	22 (13)	
LVEF < 35%	5 (5)	22 (13)	
Not documented	29 (32)	49 (29)	0.714
Primary cardiomyopathies, n (%)	11 (12)	16 (10)	
Dilated cardiomyopathy	5 (5)	11 (7)	0.712
Hypertrophic cardiomyopathy	0 (0)	5 (3)	0.200
Arrhythmogenic right ventricular dysplasia	6 (7)	0 (0)	**0.001**
Noncompaction cardiomyopathy	0 (0)	0 (0)	–
Restrictive cardiomyopathy	0 (0)	0 (0)	–
Channelopathies, n (%)	17 (18)	19 (12)	
Brugada syndrome	13 (14)	16 (10)	0.245
Short QT syndrome	0 (0)	0 (0)	–
Long QT syndrome	4 (4)	3 (2)	0.168
Other causes, n (%)			
Myocarditis	7 (8)	4 (2)	**0.027**
Takotsubo cardiomyopathy	0 (0)	0 (0)	**–**
High degree AV-block	1 (1)	1 (1)	0.668
Intoxication	6 (7)	4 (2)	0.064
Hyperkalemia	4 (4)	17 (10)	0.099
Hypokalemia	18 (20)	17 (10)	**0.034**
Coronary artery disease, n (%)			
Prior CAD	4 (4)	20 (12)	**0.043**
Prior AMI	1 (1)	16 (10)	**0.008**
AMI at index	10 (11)	46 (28)	**0.001**
STEMI	4 (4)	20 (12)	**0.018**
NSTEMI	6 (7)	26 (16)	**0.034**
Coronary angiography at index	26 (28)	96 (58)	**0.001**
No evidence of CAD	16 (62)	45 (47)	0.089
Evidence of CAD	10 (38)	51 (53)	0.184
1-vessel	10 (100)	31 (61)	**0.003**
2-vessel	0 (0)	15 (29)	
3-vessel	0 (0)	5 (10)	
Intracoronary thrombus	5 (19)	13 (13)	0.455
CABG	0 (0)	2 (2)	0.458
CTO	0 (0)	9 (9)	0.105
PCI	10 (100)	43 (84)	0.564
RCA	5 (50)	14 (30)	0.384
LMT	0 (0)	0 (0)	–
LAD	5 (50)	23 (50)	1.000
RIM	0 (0)	0 (0)	–
RCX	0 (0)	9 (20)	**0.023**
Sent to CABG	0 (0)	0 (0)	–
Electrophysiological examination, n (%)	45 (49)	68 (41)	0.203
VT ablation	14 (31)	18 (26)	0.175
Presence of ICD at discharge, n (%)	35 (38)	63 (38)	0.959
Type of ICD, n (%)			
ICD	31 (89)	58 (92)	0.788
CRT-D	0 (0)	1 (2)	
s-ICD	4 (11)	4 (6)	
ICD programming, BPM, median (IQR)			
VT detection threshold	191 (176–205)	171 (167–176)	0.735
VF detection threshold	231 (231–240)	176 (167–176)	**0.014**

Bold type indicates statistical significance *p* < 0.05

AV-block, atrioventricular block; BPM, beats per minute; CABG, Coronary artery bypass graft; CAD, coronary artery disease; CTO, coronary chronic total occlusion; ICD, implantable cardioverter-defibrillator. LAD, left anterior descending artery; LMT, left main trunk, LVEF, left ventricular ejection fraction; NSTEMI, non–ST-segment elevation myocardial infarction; PCI, percutaneous coronary intervention; RCA, right coronary artery; RCX, ramus circumflexus; RIM, ramus intermedius; STEMI, ST-Segment elevation myocardial infarction; VF, Ventricular fibrillation; VT, Ventricular tachycardia

### Distribution of coronary angiography and CAD

**Fig. 1:**
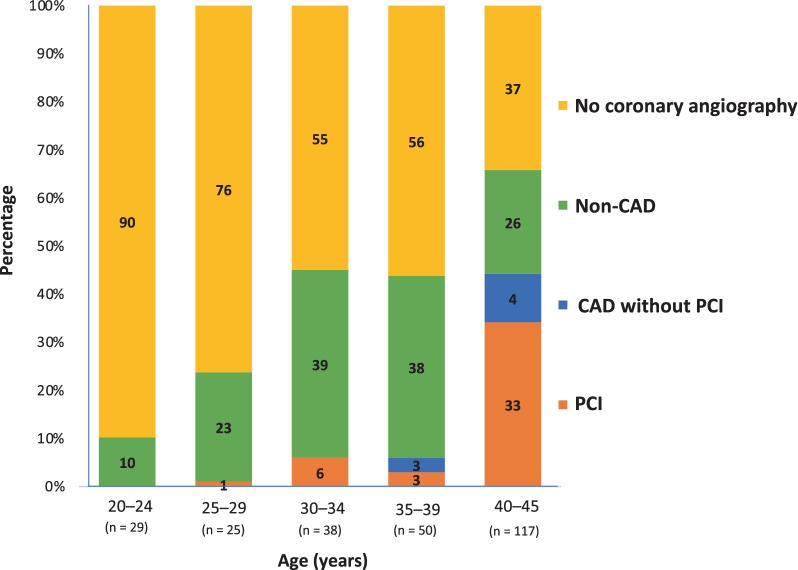
Bar diagram depicting coronary angiography rates, relevant CAD, and need for PCI according to age

Figure 1 outlines the rates of coronary angiography and CAD with and without the need for PCI according to further pre-specified age groups: 20–24 years old (n = 29), 25–29 years old (n = 25), 30–34 years old (n = 38), 35–39 years old (n = 50), and 40–45 years old (n = 117). Rates of coronary angiography, CAD, and PCI increased with age. In the youngest subgroup, only 10% underwent coronary angiography without evidence of CAD. Coronary angiography was performed in 63% of patients 40–45 years old; 37% of 40–45-year-old patients had CAD, and 33% CAD with the need for PCI.

### Cardiac diseases underlying ventricular tachyarrhythmias

**Fig. 2:**
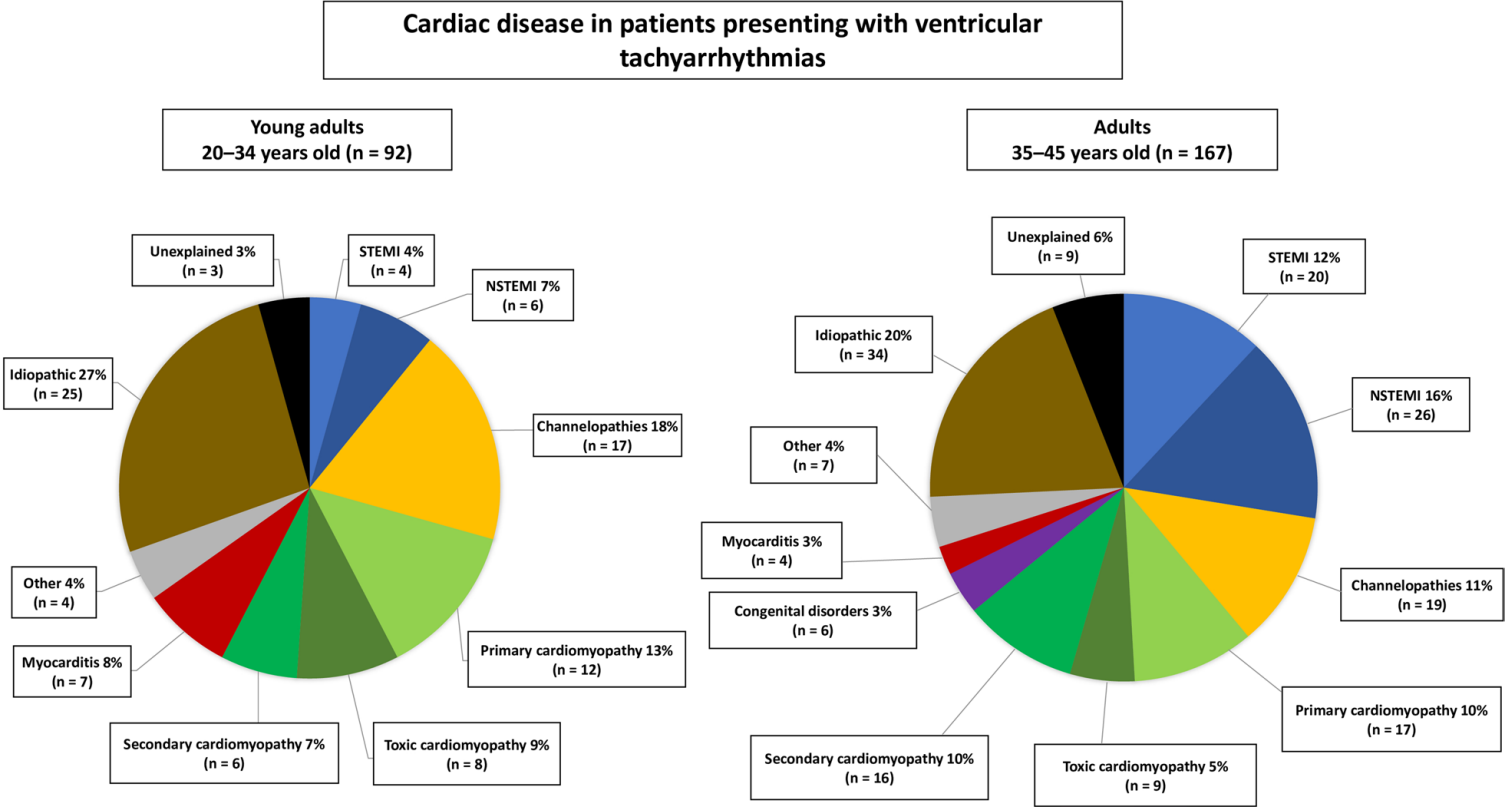
Cardiac diseases underlying VTA in young adults (left chart) and adults (right chart)

Figure 2 shows the distribution of underlying cardiac diseases in young adults (n = 92) and adults (n = 167) presenting with ventricular tachyarrhythmias at index hospitalization. In young adults (left chart), idiopathic disease (27%, n = 25) and channelopathies (18%: Brugada syndrome n = 13, long QT syndrome n = 4) were most common, followed by primary cardiomyopathies (13%: DCM n = 5, ARVD n = 7). A total 11% (n = 10) of young adults suffered from AMI (STEMI n = 4, NSTEMI n = 6). Toxic cardiomyopathies were found in 9% of patients (drug abuse n = 6, chemotherapy-induced cardiomyopathy n = 1, laxative abuse with consecutive hypokalemia n = 1). Secondary cardiomyopathies were documented regularly (7%: sarcoidosis n = 3, Friedreich-ataxia n = 1, septic cardiomyopathy n = 1, post-partum cardiomyopathy n = 1). Myocarditis affected 8% of patients (n = 7). Another 4% had other causes documented (WPW syndrome and FBI tachycardia n = 2, chronic diarrhea with hypokalemia n = 1, pulmonary embolism n = 1). Finally, 3% (n = 3) were unexplained, as these patients died within 24 h of hospital admission.

In adults (Fig. 2, right chart), the most frequent cardiac disease associated with ventricular tachyarrhythmias was AMI (28%: STEMI n = 20, NSTEMI n = 26), followed by idiopathic disease (20%), channelopathies (11%: Brugada syndrome n = 16, long QT syndrome n = 3), and primary cardiomyopathies (10%: DCM n = 11, HCM n = 5, lamin mutation n = 1). A total 10% (n = 16) of adults suffered from secondary cardiomyopathies (ischemic cardiomyopathy n = 10, septic cardiomyopathy n = 3, sarcoidosis n = 2, post-partum cardiomyopathy n = 1) and 5% from toxic cardiomyopathies (drug abuse n = 5, chemotherapy-induced cardiomyopathy n = 2, alcohol intoxication with hypokalemia n = 1, alcohol intoxication with hypoglycemia n = 1). Congenital disorders (tetralogy of Fallot n = 4, Marfan syndrome and aortic dissection n = 1, Turner syndrome and stenosis of aortic isthmus n = 1) were found in 3% of patients. Another 3% (n = 4) suffered from myocarditis, and 4% from other causes (pulmonary embolism n = 1, obstructive respiratory failure and hypoxia due to asthma n = 1, high-voltage accident n = 1, perioperative condition [orthopedic and gynecological surgery, surgical oncology (n = 3)], decompensated liver cirrhosis with hypokalemia n = 1). Of adult patients, 6% were unexplained, as they died within 24 h of hospital admission.

### Cardiac diseases in all patients undergoing CPR

**Fig. 3:**
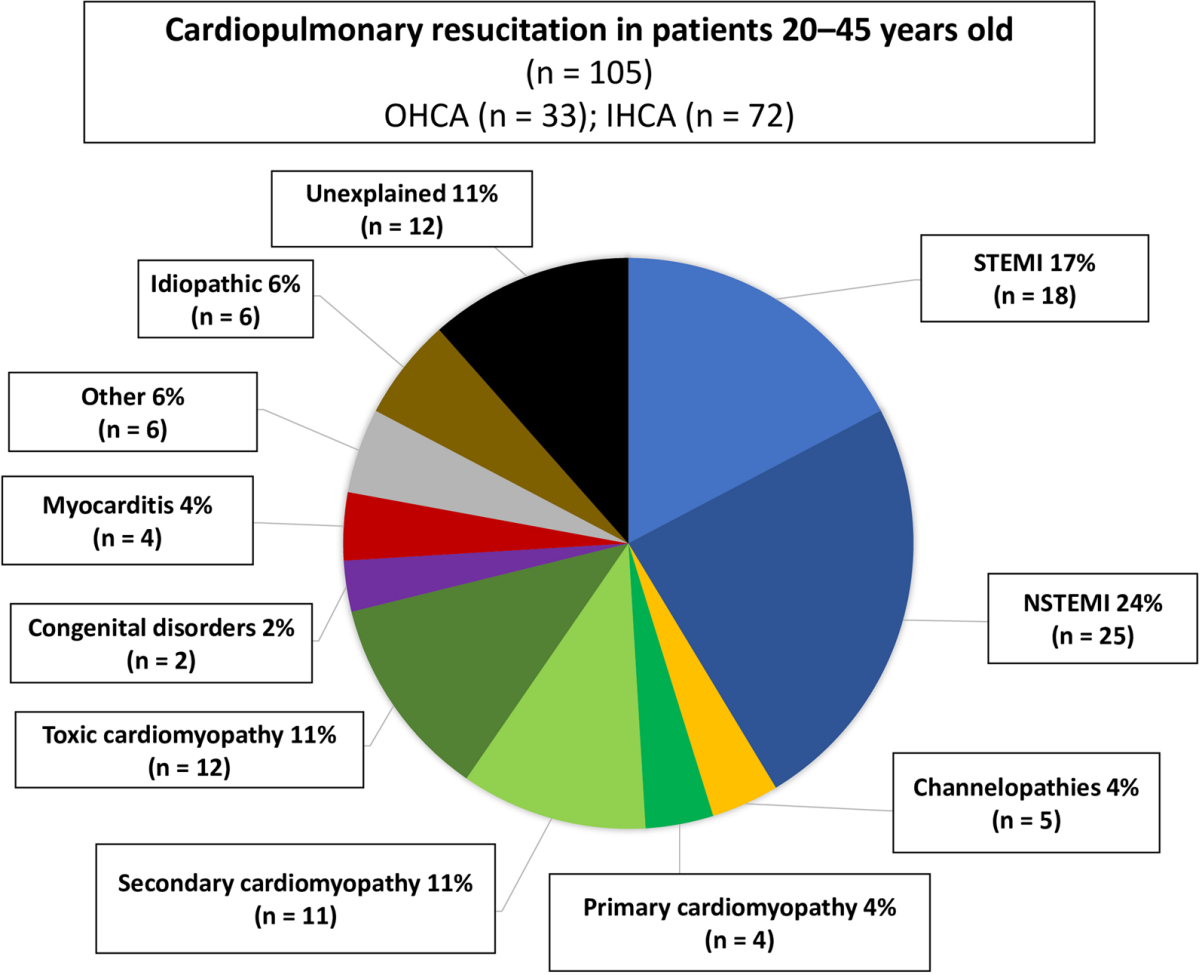
Cardiac diseases in patients who received CPR due to VTA at index hospitalization

Figure 3 shows cardiac diseases of all patients undergoing CPR (41%, n = 105); 31% of these patients experienced OHCA and 69% IHCA. The most frequent cardiac disease was AMI, with 41% (17% STEMI n = 18, 24% NSTEMI n = 25) followed by 11% for secondary cardiomyopathies (septic cardiomyopathy n = 5, ICMP n = 4, post-partum cardiomyopathy n = 2). Another 11% of patients had toxic cardiomyopathy (drug abuse n = 8, laxative abuse with hypokalemia n = 1, alcohol intoxication with hypoglycemia n = 1, chemotherapy-induced cardiomyopathy n = 2), and 6% had idiopathic ventricular tachyarrhythmia. Channelopathies (Brugada syndrome n = 1, long QT syndrome n = 4), primary cardiomyopathies (DCM n = 2, HCM n = 1, lamin mutation n = 1), and myocarditis were each found in 4% of patients. Congenital disorders affected 2% of patients (Marfan syndrome and aortic dissection n = 1, Turner syndrome and stenosis of aortic isthmus n = 1), and 6% had other causes (high-voltage accident n = 1, pulmonary embolism n = 2, perioperative oncological and gynecological surgery n = 2, WPW syndrome and FBI tachycardia n = 1). Finally, 11% of patients were unexplained, as they died within 24 h of hospitalization.

### Primary and secondary endpoints

**Fig. 4:**
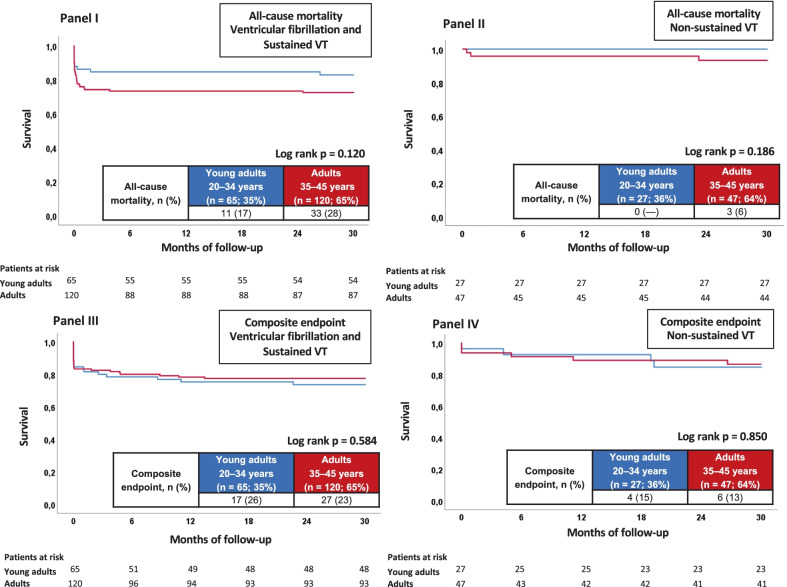
Kaplan–Meier analysis for the primary endpoint of all-cause mortality at 2.5 years and the composite arrhythmic endpoint in patients with ventricular fibrillation and sustained VT (panel I and III) and in patients with non-sustained VT (panel II and IV)

Young adults appeared to show better survival at 2.5 years than adults, but this effect failed to meet statistical significance. (12% vs. 21%; HR = 1.862, 95% CI 0.948–3.658, p = 0.058, log-rank p = 0.063) (Table 3). All-cause mortality at index hospitalization was 10% vs. 18% (Table 3). Kaplan–Meier analysis was performed in the subgroups of patients with VF and sustained VT (Fig. 4, panel I and III) and those with non-sustained VT (Fig. 4, panel II and IV). Young adults and adults in both subgroups had similar results for the primary endpoint of all-cause mortality at 2.5 years and the composite arrhythmic endpoint (Fig. 4). Notably, all young adults presenting with non-sustained VT survived the follow-up of 2.5 years.

**Table 3:** Primary and secondary endpoints

Characteristic	Young adults 0–34 years old (n = 92; 36%)	Adults 35–45 years old (n = 167; 64%)	*p* value
Primary endpoint, n (%)			
All-cause mortality at 2.5 years	11 (12)	36 (21)	0.058
Secondary endpoints, n (%)			
Cardiac death at 24 h	8 (9)	18 (11)	0.593
All-cause mortality at index hospitalization	9 (10)	30 (18)	0.078
All-cause mortality after index hospitalization	4 (4)	15 (9)	0.171
Composite endpoint at 2.5 years (Cardiac death at 24 h, recurrent ventricular tachyarrhythmias, appropriate ICD therapies)	21 (23)	33 (20)	0.561

ICD, implantable cardioverter-defibrillator

### Cox regression analyses in young adults and adults

Cox regression analyses were performed to find predictors of long-term all-cause mortality in young adults and adults. After multivariate adjustment, only the presence of LVEF < 35% (HR = 33.590, 95% CI 2.395–471.404, p = 0.009) was associated with increased all-cause mortality at 2.5 years in young adults (Table 4). No further predictors for the primary endpoint and the composite arrhythmic endpoint were found (Table 4).

**Table 4:** Multivariate Cox regression models

Variables	All-cause mortality at 2.5 years					
	Young adults 20–34 years old 20–34 years old (n = 92; 36%)			Adults 35–45 years old 35–45 years old (n = 167; 64%)		
	HR	95% CI	*p* value	HR	95% CI	*p* value
Males	0.095	0.007–1.240	0.273	1.135	0.299–4.310	0.852
Chronic kidney disease	1.064	0.090–1.064	0.961	2.492	0.822–7.553	0.106
LVEF < 35%	33.590	2.395–471.404	**0.009**	2.296	0.822–7.553	0.164
AMI	2.073	0.149–28.910	0.588	1.053	0.364–3.047	0.924
Atrial fibrillation	11.090	0.618–199.219	0.102	1.469	0.364–3.047	0.459
Ventricular fibrillation	3.840	0.322–44.354	0.281	2.975	0.904–9.796	0.073

Variables	Composite endpoint at 2.5 years (cardiac death at 24 h, recurrent ventricular tachyarrhythmias, appropriate ICD therapies)					
	Young adults 20–34 years old			Adults 35–45 years old		
	HR	95% CI	*p* value	HR	95% CI	*p* value
Males	0.526	0.179–1.543	0.242	0.763	0.278–2.095	0.599
Chronic kidney disease	1.349	0.368–5.025	0.645	1.532	0.539–4.351	0.423
LVEF < 35%	0.894	0.114–7.013	0.915	1.739	0.594–5.096	0.313
AMI	0.830	0.084–8.198	0.873	0.741	0.234–2.349	0.611
Atrial fibrillation	1.354	0.165–11.127	0.778	0.995	0.348–2.848	0.922
Ventricular fibrillation	0.443	0.105–1.875	0.296	0.765	0.293–1.999	0.584

Bold type indicates statistical significance *p* < 0.05

AMI, acute myocardial infarction; CI, confidence interval; HR, Hazard ratio; LVEF, left ventricular ejection fraction

## Discussion

This study retrospectively investigated consecutive patients between 20- and 45-years old presenting at hospital admission with VTA and at high risk of SCD.

There is a relevant knowledge gap regarding the rates of underlying cardiac diseases in young adults presenting with VTA, and victims of SCD [2, 16]. While recent studies usually have focused on patients with documented SCD, the present study focused instead on high-risk patients with documented VTA and potentially endangered by SCD.

Maron et al. recently reported on the US National Registry of Sudden Death in Athletes, including 1,049 cases of SCD in young athletes, presumably caused by cardiac disease [3]. HCM was the most common cause (36%), followed by coronary anomalies (17%), which includes the atypical origin of the sinoatrial nodal artery [3]. Other cardiac diseases, including CAD, myocarditis, and channelopathies, accounted for less than 6% of cases [3]. Based on evidence like this, HCM is widely believed to be the most common cause of SCD in young athletes [1, 4]. However, young athletes represent a very select cohort and do not represent the general young adult population [4]. In contrast, the present study, which used a non-specified, general population, showed no cases of SCD caused by HCM in young adults, and found HCM caused only 3% of SCD cases in adults. However, more than one-third of young adults undergoing coronary angiography had evidence of relevant CAD with the need for PCI.

Allan et al. investigated a web-based multicentric database. In this observational cohort, 608 young adults with OHCA and presumed cardiac disease were investigated [1]. The presence of CVRF increased with age. At least 50% of young adults and 65% of adults suffered from at least one cardiovascular risk factor. CAD affected 6% of young adults and 34% of adults. In the entire study cohort, 29% suffered from structural heart diseases, and 16% had unexplained deaths [1].

The present study demonstrates that CVRF increase with age in patients presenting with VTA. However, CVRF and CAD were also found in 10% of young adults. Of the patients who received CPR, 41% had AMI. The present study confirms the results of prior studies, demonstrating that the rate of CAD is higher and the need for PCI is more common than expected in adults, as well as in young adults presenting with ventricular tachyarrhythmias at index hospitalization. Although CAD and AMI were less common in young adults, the index hospitalization coronary angiography rate of 28% was high. CAD affected 38% of young adults, of whom 100% needed PCI. In adults, even more patients (58%) underwent coronary angiography at index hospitalization. CAD affected 53%, of whom 84% needed PCI.

Jayaraman et al. investigated circumstances, resuscitation outcomes, CVRF, and physical activity as potential triggers of SCD in 186 patients between 5 and 35 years old [2]. The authors found that only 14% of SCD cases were associated with physical activity. In contrast, the overall most common SCD-related condition was sudden arrhythmic death syndrome, which affected 31% of patients, followed by CAD at 22%, and HCM at 14% [2]. Sudden arrhythmic death syndrome was assigned to cases with no identified cause of SCD despite autopsy (65% of patients) or complete medical workup [2]. Notably, the authors reported an unexpectedly high rate of CVRF, with at least one factor in 58% of cases [2].

Wisten et al. found similar results for 552 SCD victims between the ages of 1 and 35. A forensic autopsy was performed on all patients. The most frequent etiology of SCD, affecting 31% of patients, was sudden arrhythmic death syndrome, followed by CAD at 15%, myocarditis at 14%, and unspecified cardiomyopathy at 12% [15]. Only 5% of patients had HCM, and 4% had ARVD. Notably, sudden arrhythmic death syndrome was defined as the condition of a structurally normal heart at the histological examination with inconclusive autopsy and toxicology investigations by excluding non-cardiac etiologies [15].

The present study showed a relevant rate of idiopathic and unexplained ventricular tachyarrhythmias, which might potentially reflect the number of patients with sudden arrhythmic death syndrome that Jayaraman et al. and Wisten et al. found in their studies. CAD was the second most common cause of SCD in both Wisten et al. and Jayaraman et al., confirming the higher CAD rate in young adults and adults reported in the present study [1, 15].

The present study evaluates the rare phenomenon of VTA in patients between 20 and 45 years old. Since prior studies have investigated heterogeneous cohorts of patients who have received CPR, or SCD victims, the present study’s major strength is the earlier inclusion of patients with VTA who are endangered by potential SCD. Despite high rates of idiopathic disease, CAD and AMI were unexpectedly a more common cause of ventricular tachyarrhythmias and CPR.

### Study limitations

This observational and retrospective, registry-based analysis provides a realistic depiction of the health care provided to consecutive high-risk patients presenting with VTA. The lost-to-follow-up rate for the evaluated endpoint of all-cause mortality was minimal. All patients diagnosed with VT, SVT and NSVT at our institution were included. Unless accompanied by symptoms, the identification of NSVT depends on the frequency of testing and, therefore, may be underestimated in the present study.

A stepwise statistical approach was used to control heterogeneity within the study population. This included multivariate adjustment for several important comorbidities and risk factors and Kaplan–Meier analysis according to different types of VTA. However, the outcomes of patients presenting with ventricular tachyarrhythmias may have been influenced by unmeasured confounders. This study did not include patients who passed away following OHCA or those who were not transferred to the heart center. Individual cardiologists documented all clinical data during routine clinical care. These cardiologists were blinded to final data analyses, thereby obviating an independent clinical event committee. Unfortunately, genetic testing for hereditary VTA was incomplete in the present study. Therefore, high rates of idiopathic VTA might be overstated. Further studies should include genetic testing for hereditary VTA. Further studies are also needed to reevaluate the impact of age on the prognosis of patients with ventricular tachyarrhythmias who are 45 years old or younger and to reevaluate predictors for long-term all-cause mortality in those patients.

## Conclusion

The present study emphasizes the increasing rate of CAD and need for PCI as a potential and unexpectedly more common cause of VTA in patients 45 years old and younger. Among young adults, idiopathic VTA was most common, followed by channelopathies, whereas primary cardiomyopathies and AMI were less common. In contrast, adults more often had AMI and less often idiopathic disease, channelopathies and primary cardiomyopathies. CPR was performed on 41% of all patients, for whom AMI was most frequent (41%). Young adults and adults had comparable survival at index hospitalization and after 2.5 years.

## Data Availability

The authors can confirm that all relevant data are included in the article.

## Abbreviations

CAD: Coronary artery disease
CKD: Chronic kidney disease
CPR: Cardiopulmonary resuscitation
ICD: Implantable cardioverter-defibrillator
LVEF: Left ventricular ejection fraction
NSTEMI: Non-ST-segment elevation myocardial infarction
NSVT: Non-sustained ventricular tachycardia
STEMI: ST-segment elevation myocardial infarction
SVT: Sustained ventricular tachycardia
VTA: Ventricular tachyarrhythmia
VT: Ventricular tachycardia
VF: Ventricular fibrillation
